# A Machine Learning-Modified Novel Nomogram to Predict Perioperative Blood Transfusion of Total Gastrectomy for Gastric Cancer

**DOI:** 10.3389/fonc.2022.826760

**Published:** 2022-04-11

**Authors:** Jiawen Zhang, Linhua Jiang, Xinguo Zhu

**Affiliations:** Department of General Surgery, The First Affiliated Hospital of Soochow University, Suzhou, China

**Keywords:** gastric cancer, total gastrectomy, blood transfusion, nomogram, machine learning

## Abstract

**Background:**

Perioperative blood transfusion reserves are limited, and the outcome of blood transfusion remains unclear. Therefore, it is important to prepare plans for perioperative blood transfusions. This study aimed to establish a risk assessment model to guide clinical patient management.

**Methods:**

This retrospective comparative study involving 513 patients who had total gastrectomy (TG) between January 2018 and January 2021 was conducted using propensity score matching (PSM). The influencing factors were explored by logistic regression, correlation analysis, and machine learning; then, a nomogram was established.

**Results:**

After assessment of the importance of factors through machine learning, blood loss, preoperative controlling nutritional status (CONUT), hemoglobin (Hb), and the triglyceride–glucose (TyG) index were considered as the modified transfusion-related factors. The modified model was not considered to be different from the original model in terms of performance, but is simpler. A nomogram was created, with a C-index of 0.834, and the decision curve analysis (DCA) demonstrated good clinical benefit.

**Conclusions:**

A nomogram was established and modified with machine learning, which suggests the importance of the patient’s integral condition. This emphasizes that caution should be exercised regarding transfusions, and, if necessary, preoperative nutritional interventions or delayed surgery should be implemented for safety.

## Introduction

Gastric cancer (GC) remains a significant health issue worldwide, and surgery is still the preferred treatment method. GC is the third leading cause of cancer-related deaths worldwide (1, 2). The increasing prevalence of upper and middle tumors, as well as the larger extent of resection and the difficulty of anastomosis in total gastrectomy (TG), has prompted scholars to focus on research related to TG (3, 4).

A significant proportion of patients requires blood transfusion during the perioperative period of gastrointestinal surgery, which has become a common treatment (4, 5). The preoperative nutritional status, tumor consumption, and intraoperative hemorrhage determine the need for perioperative blood transfusion. Studies have shown that whether it is gastrectomy or colorectal surgery, blood transfusion is a risk factor affecting the prognosis of patients (6). However, only a few studies have focused on the influence of blood transfusion on patients’ short-term outcomes. Additionally, there is misuse or neglect of blood transfusion in addition to the variability of patients’ conditions. Meanwhile, studies have indicated that the choice of blood transfusion depends on the details of the surgical procedure, preoperative hemoglobin (Hb), and tumor stage, among others, and blood transfusion therapy is still clinically significant for critically ill patients (7).

In the current environment affected by coronavirus disease 2019 (COVID-19), perioperative blood transfusion reserves are extremely limited; therefore, it is important to develop plans for perioperative blood transfusions. No study has provided guidance on the prediction of blood transfusion outcomes. Machine learning is an emerging technology for analyzing data, improving clinical decision-making, and establishing predictive models (8–10).

This study used readily available clinical data to build a predictive model identifying patients at risk of perioperative transfusion during TG. Furthermore, machine learning was used to simplify the model to obtain a streamlined and accurate prediction model. This model allows clinicians to actively prepare blood resources, advance preoperative interventions, and ensure the clinical safety of patients.

## Materials and Methods

### Study Patients

**Figure 1:**
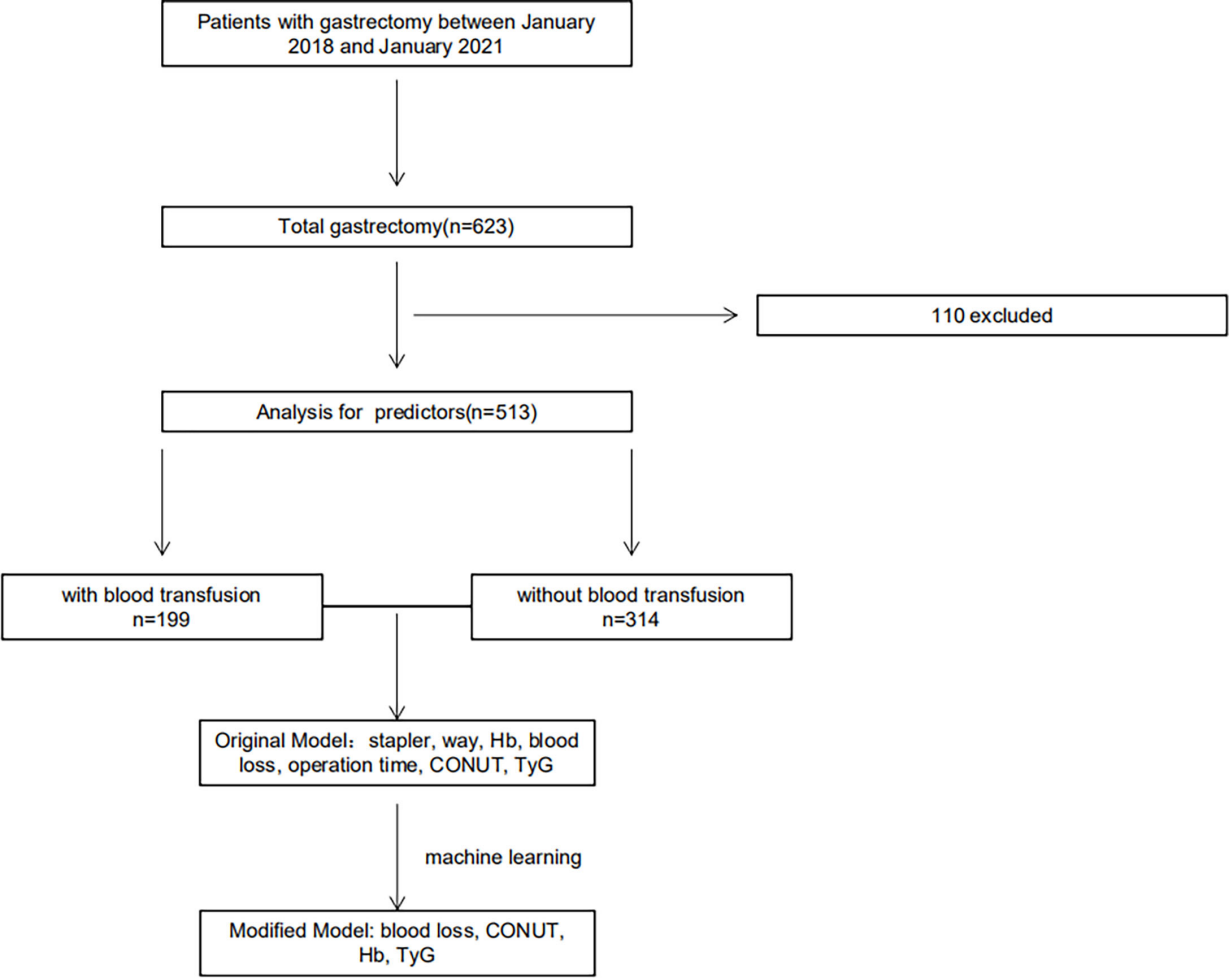
Flowchart of the study. A total of 513 patients were included in the final study for perioperative transfusion-related analyses.

This retrospective study collected the data of GC patients undergoing TG with D2 dissection at The First Affiliated Hospital of Soochow University from January 2018 to January 2021. **Figure 1** shows the patient selection process. A total of 513 patients were included according to the inclusion and exclusion criteria. The Ethics Committee of the First Affiliated Hospital of Soochow University approved this study. The protocol of this retrospective observational study involved minimal risk and did not present a threat to the health of the subjects.

### Inclusion and Exclusion Criteria

The inclusion criteria of this study were as follows: 1) preoperative or postoperative pathology consistent with gastric malignancy and 2) patients having undergone TG with D2 lymphadenectomy. The exclusion criteria were: 1) stage IV proven in any form; 2) palliative surgery; 3) combined organ removal; and 4) neoadjuvant chemotherapy.

### Data Collection

In this study, blood transfusion, mainly perioperative blood transfusion, was defined as the transfusion from the first admission to discharge. Moreover, perioperative blood transfusion was mainly determined by the physician according to the patient’s condition, with no specific criteria for blood transfusion and no strict regulations to regulate transfusion thresholds.

The following baseline data were collected: age, gender, body mass index (BMI), Hb at initial evaluation after admission and before discharge, preoperative controlling nutritional status (CONUT) score, preoperative triglyceride–glucose (TyG) index, preoperative prognostic nutritional index (PNI), preoperative prealbumin, and preoperative albumin (ALB). Data on the stapler used (line or circle stapler), surgical technique (open surgery or laparoscopic surgery), blood loss, operation time, nutrition feeding tube, cost, and hospital stay were also obtained. Simultaneously, concerning tumors, data on tumor size, T stage, N stage, number of lymph nodes, vascular invasion, and nerve invasion were collected. At the first admission, information on the preoperative status was extracted. Tumor size was measured with a combination of intraoperative conditions and postoperative pathology according to the long and short diameters of the tumor.

Data on early postoperative complications higher than grade II were collected according to the Clavien–Dindo classification within 30 days after surgery. Clinical symptoms and signs, CT, and endoscopy were used to diagnose the complications.

### Statistical Analysis

Patients were categorized into two groups based on receiving or not receiving a blood transfusion. The patients in the two groups were matched using 1:1 propensity score matching (PSM). The age, gender, BMI, and the long and short diameters of the tumor were used to calculate the individual propensity score; the caliper value was set to 0.01.

In this study, six types of machine learning algorithms were assessed: logistic regression (LR), decision tree learning (Tree), XGBoost (XGB), random forest (RF), gradient boosting decision tree (GBDT), and light gradient boosting machine (GBM). XGB (extreme gradient boosting) is an improvement of the GBDT. It can be used not only for classification problems but also for regression problems. This algorithm uses positive lateralization to prevent overfitting and is a relatively novel algorithm.

Univariate and multivariate logistic regressions and machine learning were used to explore the relationship between the variables and blood transfusion. The results were displayed as odds ratios (ORs) and 95% confidence intervals (CIs). The concordance index (C-index) was used to measure the differences between the performance and the predicted results of the nomogram. The C-index correctly predicted the probability of positive events in a survival prediction model through a group of randomly selected patients. Moreover, calibration curves were used to compare the predicted results of the nomogram with the actual results, while the 45° line was used as the optimal model. The data were 7:3 randomly divided into the training and testing cohorts.

Continuous variables with normal distributions were presented as means and standard deviations, and categorical variables were presented as numbers (percentages). Statistical analyses were performed using Python 3.8.5, SPSS 26.0, and R software programs. A *p* < 0.05 indicated a statistically significant difference.

## Results

### Clinicopathological Characteristics and Short-Term Outcomes of Patients

**Figure 1** presents the flowchart of patient selection in this retrospective study. In total, 199 patients who had blood transfusion and 314 without blood transfusion were included in the study. As displayed in **Table 1**, 210 patients were successfully matched after PSM, with their clinical and pathological characteristics also shown. Based on the study design, the age, gender, BMI, and the long and short diameters of tumor were compared. The histogram demonstrated rigorous matching effectiveness (**Supplementary Figure S1**). Before PSM, the long and short diameters of tumor were significantly larger in the blood transfusion group than those in the non-transfusion group. Possible biases were reduced by PSM, with no significant differences between the groups in age, gender, BMI, and in the tumor long and short diameters after PSM.

**Table 1:** Comparison of clinical and pathological data of the patients with PSM.

Variable	All patients (n=513)			Patients after matching (n=210)		
	With blood transfusion (n=199)	Without blood transfusion (n=314)	P	With blood transfusion (n=105)	Without blood transfusion (n=105)	P
Age (years)	66.21	66.34	0.9055	66.49	67.07	0.7177
Gender			0.1477			0.9999
Male	140	240		73	74	
Female	59	74		32	31	
BMI	22.52	23.09	0.0890	23.03	22.77	0.6005
Tumor long diameter	5.753	4.623	0.0003	4.449	4.386	0.8203
Tumor short diameter	4.182	3.483	0.0025	3.376	3.404	0.9068

BMI, body mass index.

**Table 2** presents the differences in the short-term hospitalization results of the two groups regarding blood transfusions. There were no significant differences between the two groups regarding postoperative Hb, Hb changes, and percentages both before and after PSM (*p* > 0.05). In terms of postoperative recovery, the hospital stay in the non-transfusion group was shorter than that in the blood transfusion group. Moreover, the expenditures in the non-transfusion group were lower in terms of the total cost of hospitalization and disposable items for surgery (*p* < 0.05). The blood transfusion group exhibited a lower incidence of early postoperative complications before PSM (*p* = 0.0115), and there was a nearly significant difference between the two groups after using PSM to remove possible bias (*p* = 0.0714).

**Table 2:** Short-term outcomes of patients who had total gastrectomy (TG) before and after propensity score matching (PSM).

Variable	All patients (*n* = 513)			Patients after matching (*n* = 210)		
	With blood transfusion (*n* = 199)	Without blood transfusion (*n* = 314)	*p*-value	With blood transfusion (*n* = 105)	Without blood transfusion (*n* = 105)	*p*-value
Postoperative Hb	113.4	106.7	0.0006	108.7	110.6	0.4631
Hb change	−4.778	−9.257	0.0494	−7.986	−10.77	0.4608
Hb change (%)	0.69	−5.12	0.0232	−2.05	−6.13	0.3227
Hospital stay	16.79	15.16	0.0039	16.87	14.90	0.0252
One-time consumables for surgery	31,620	28,515	0.0022	32,495	27,388	0.0015
Total cost of hospitalization	74,638	63,947	<0.0001	76,773	61,939	<0.0001
Early postoperative complications			0.0115			0.0714
No	169	290		90	98	
Yes	30	24		15	7	

Hb, hemoglobin.

### Risk Factors Associated With Blood Transfusion

**Table 3** shows the differences in the clinical data between the two groups, including nutritional indicators, pathological data, and surgical outcomes. After PSM, the blood transfusion group had lower Hb (*p* < 0.0001) and higher TyG (*p* = 0.0188) than the non-transfusion group, with the blood transfusion group exhibiting a worse CONUT score (*p* < 0.0001). Regarding the nutritional indicators, such as PNI, prealbumin, and ALB, there was no statistical difference between the two groups after PSM in the pathological indicators, including vascular invasion, nerve invasion, T stage, and N stage.

**Table 3:** Comparison of the clinical, pathological, and surgical data of patients.

Variable	All patients (*n* = 513)			Patients after matching (*n* = 210)		
	With blood transfusion (*n* = 199)	Without blood transfusion (*n* = 314)	*p*-value	With blood transfusion (*n* = 105)	Without blood transfusion (*n* = 105)	*p*-value
Preoperative Hb	104.2	126.3	<0.0001	101.3	124.5	<0.0001
CONUT score			<0.0001			<0.0001
0–1	33	139		15	45	
2–4	75	135		34	49	
5–8	64	37		37	9	
9–12	27	3		19	2	
TyG	8.565	8.234	<0.0001	8.476	8.268	0.0188
PNI	44.39	45.18	0.5316	48.17	45.30	0.2625
Prealbumin	186.7	204.2	0.0500	205.1	201.8	0.7171
ALB	35.63	37.83	0.0006	37.55	37.72	0.8589
Vascular invasion			0.2657			0.5487
Yes	82	114		34	30	
No	117	200		71	75	
Nerve invasion			0.7205			0.6743
Yes	83	136		42	45	
No	116	178		63	60	
T stage			0.0535			0.5363
1–2	24	58		15	12	
3–4	175	256		90	93	
N stage			0.3338			0.9370
0	52	102		33	30	
1	30	51		15	18	
2	48	72		27	27	
3	69	89		30	30	
Stapler			0.0054			0.0280
CS	142	257		81	93	
LS	57	57		24	12	
Surgical technique			<0.0001			<0.0001
Laparoscopy	66	185		36	70	
Open	133	129		69	35	
Blood loss			<0.0001			<0.0001
<200	102	246		48	87	
200–400	64	63		34	15	
>400	33	5		23	3	
Operation time	246.6	224.1	0.0097	252.6	211.8	0.0016
Nutrition tube			0.9485			0.4270
Yes	69	108		29	24	
No	130	206		76	81	
No. of total lymph nodes	29.90	21.71	0.3673	26.11	21.06	0.2907
No. of positive lymph nodes	8.13	4.867	0.3761	10.77	4.871	0.2387

Hb, hemoglobin; CONUT, controlling nutritional status; TyG, triglyceride–glucose; PNI, prognostic nutritional index; ALB, albumin; CS, circle stapler; LS, linear stapler.

Concerning surgery, a linear stapler was used more frequently, and patients in the blood transfusion group underwent more open surgery to complete the procedures (*p* < 0.05). Meanwhile, the operation time and the estimated blood loss were higher in the transfusion group compared to the non-transfusion group (*p* < 0.05). There were no significant differences between the two groups in the use of feeding tubes and the number of resected lymph nodes (*p* > 0.05).

**Figure 2:**
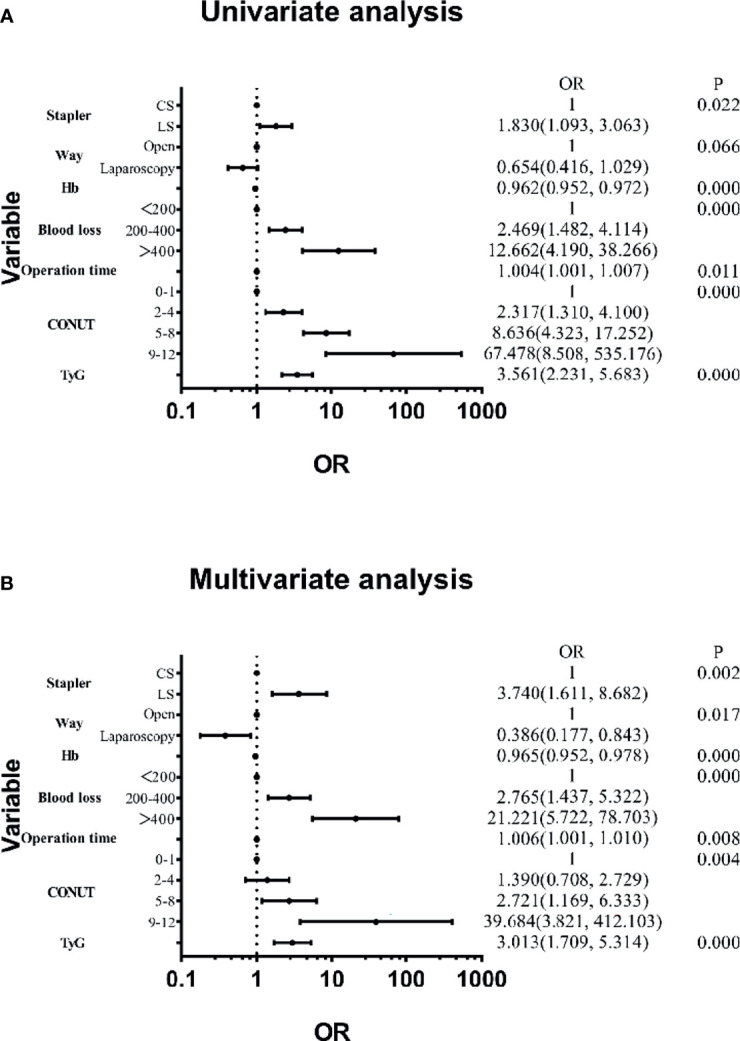
**(A)** Results of univariate analysis. **(B)** Results of multivariate analysis.

**Figure 3:**
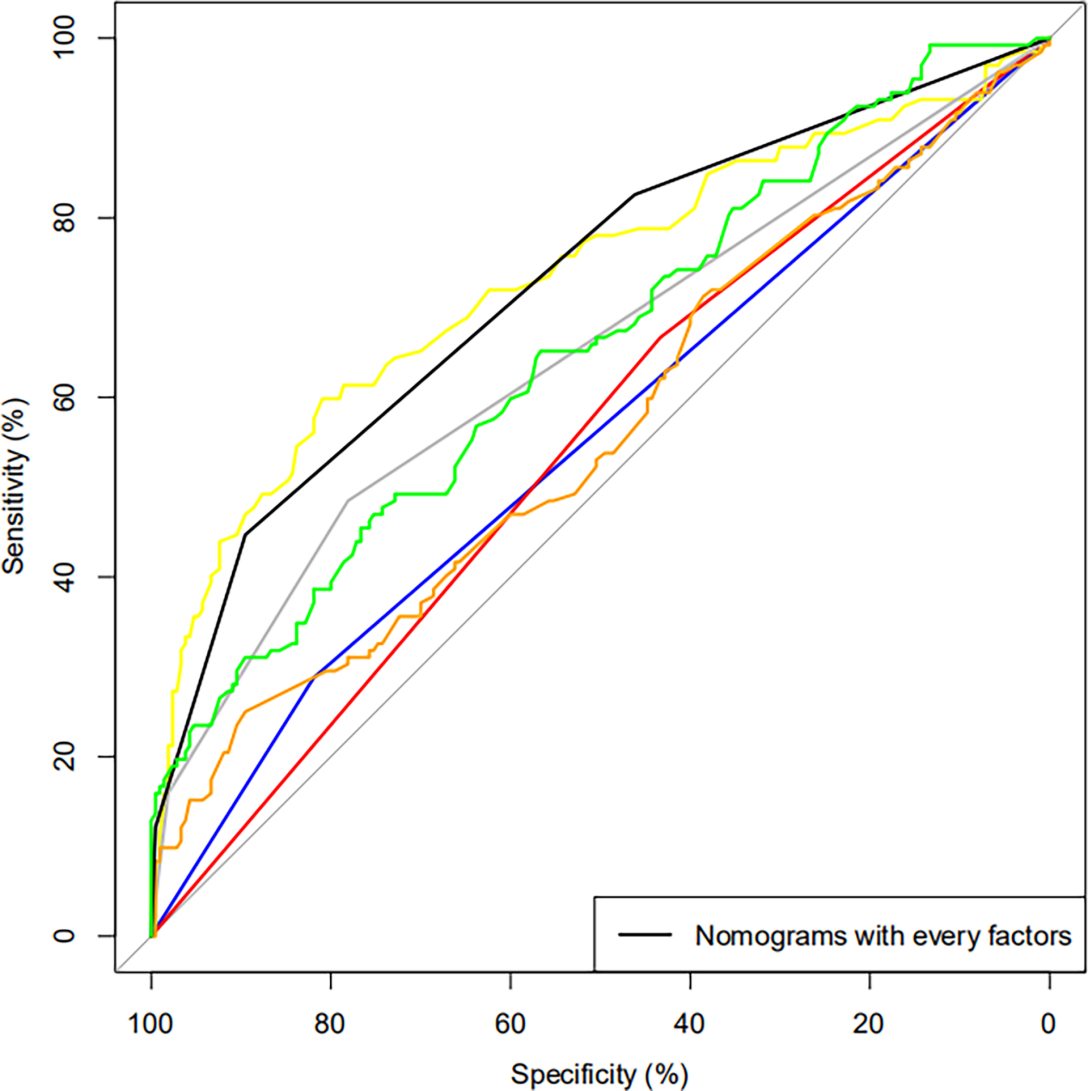
Receiver operating characteristic (ROC) curve of blood transfusion based on logistic regression for each variable. *Blue*, stapler; *red*, surgical technique; *yellow*, hemoglobin (Hb); *gray*, blood loss; *orange*, operation time; *black*, preoperative controlling nutritional status (CONUT); *green*, triglyceride–glucose (TyG).

Features were evaluated using univariate and multivariate analyses, which showed that the factors associated with blood transfusion were the stapler used, surgical technique, Hb, blood loss, operation time, CONUT, and TyG (**Figure 2**). The others were not significant risk factors. **Figure 3** further shows the area under the curve (AUC) for the stapler used, surgical technique, Hb, blood loss, operation time, CONUT score, and the TyG index.

### Improvements Based on Machine Learning

**Figure 4:**
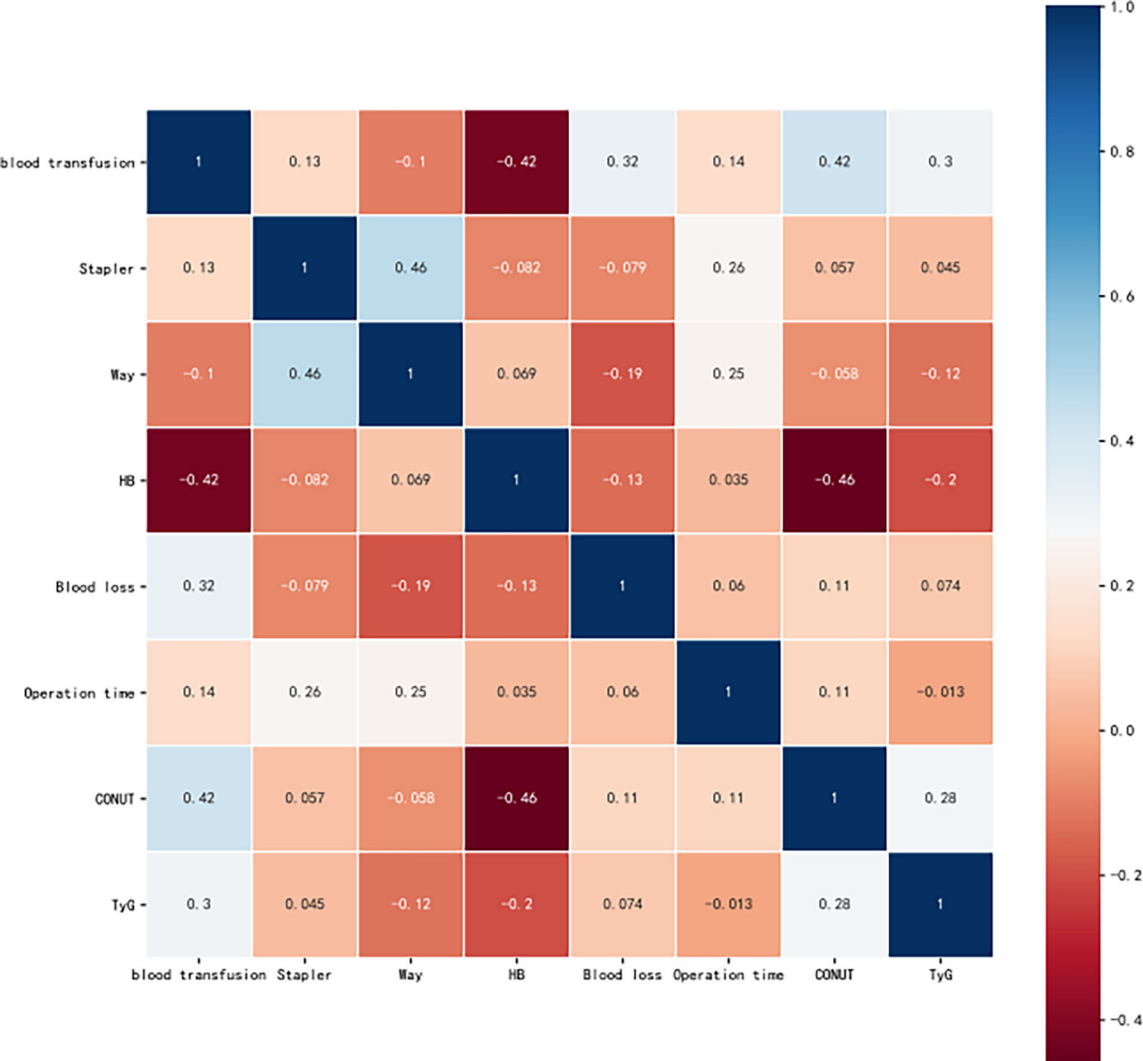
Correlation between variables.

**Figure 5:**
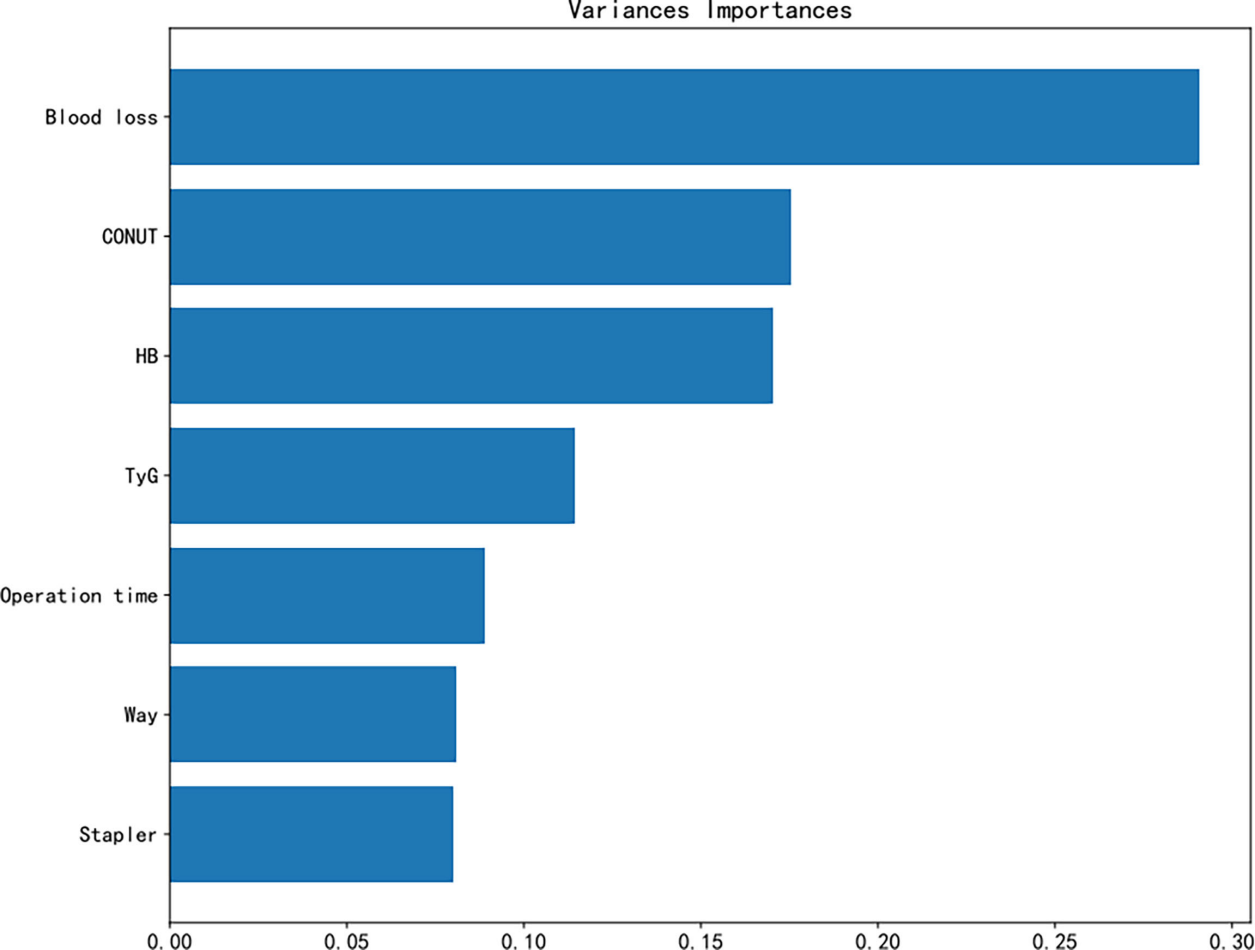
Variable importance of the features included in extreme gradient boosting (XGB) for prediction of blood transfusion.

The correlation analysis showed that the stapler used, blood loss, operation time, CONUT, and TyG were positively correlated with blood transfusion, with blood loss, CONUT, and TyG exhibiting a strong connection. Meanwhile, Hb and the surgical technique were negatively correlated with blood transfusion. Therefore, it is expected that the surgical technique and stapler used were related (**Figure 4**). To evaluate our model, we used the area under the receiver operating characteristic (AUC-ROC) curve. **Supplementary Figure S2** shows the performance of 6 machine learning algorithms. The logistic regression model performed the best (AUC = 0.879), while the decision tree performed the worst (AUC = 0.867). In addition, **Figure 5** shows the importance of seven factors in the XGB algorithm, with the order of importance from high to low: blood loss, CONUT, Hb, TyG, operation time, surgical technique, and the stapler used.

Combining logistic regression, correlation analysis, and XGB, we further screened for transfusion-related risk factors and considered blood loss, CONUT, Hb, and TyG as the main factors to modify the nomogram model.

**Figure 6:**
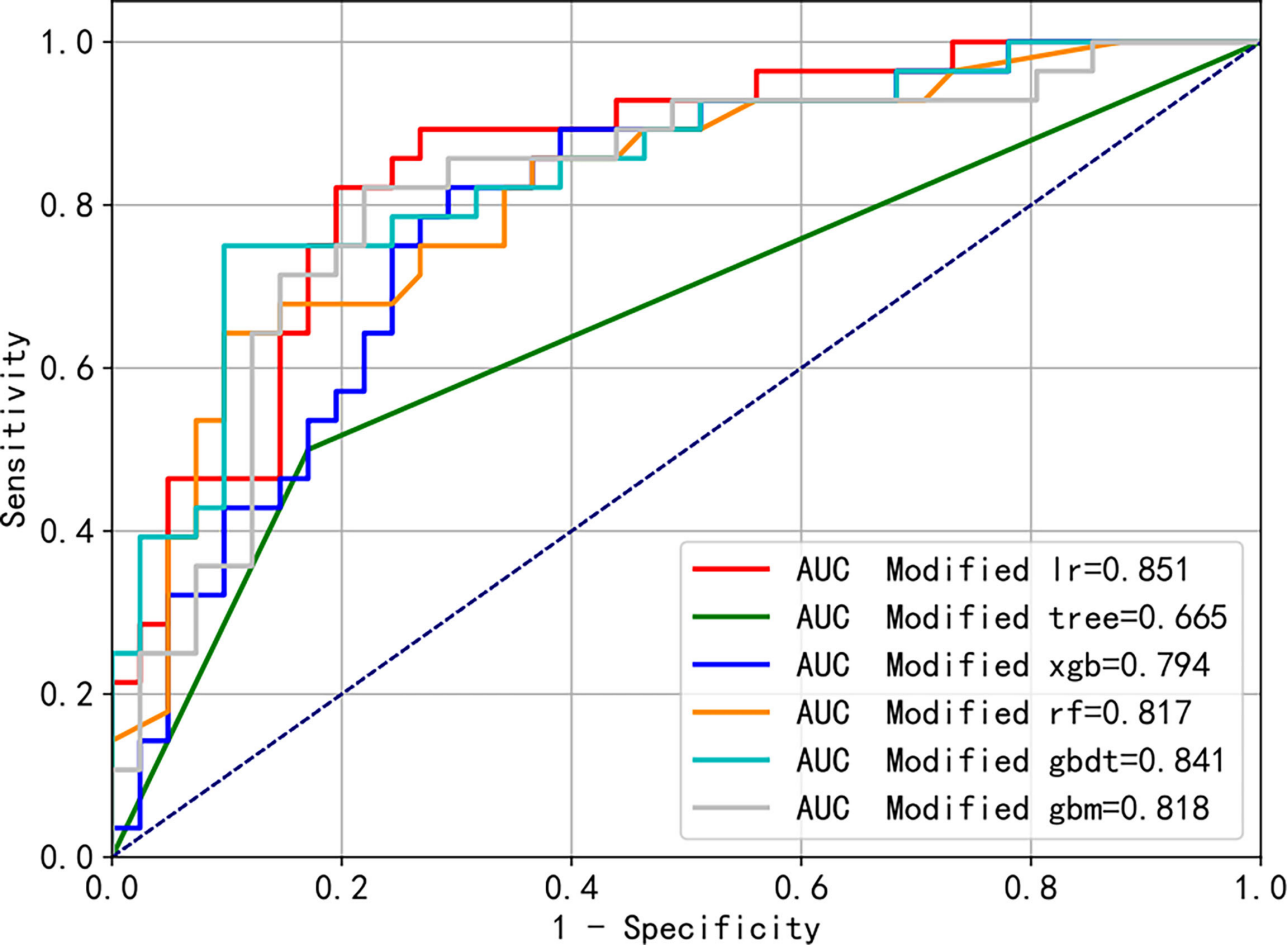
Modified receiver operating characteristic (ROC) curves of the different machine learning algorithms predicting blood transfusion.

**Figure 6** shows the AUCs of the six algorithms after improving the model, considering only the four factors, which showed a similar trend to that before the improvements. The AUCs, ranged from high to low, were as follows: logistic regression, 0.851; gradient boosting decision tree, 0.841; light gradient boosting machine, 0.818; random forest, 0.817; XGBoost, 0.794, and decision tree, 0.665. Moreover, **Supplementary Figure S3** shows the differences in the ROC curves before and after improvement using the XGB algorithm, which were not statistically different (AUC = 0.796 *vs.* 0.794, *p* = 0.478).

### Performance Assessment and Validation of the Nomogram

**Figure 7:**
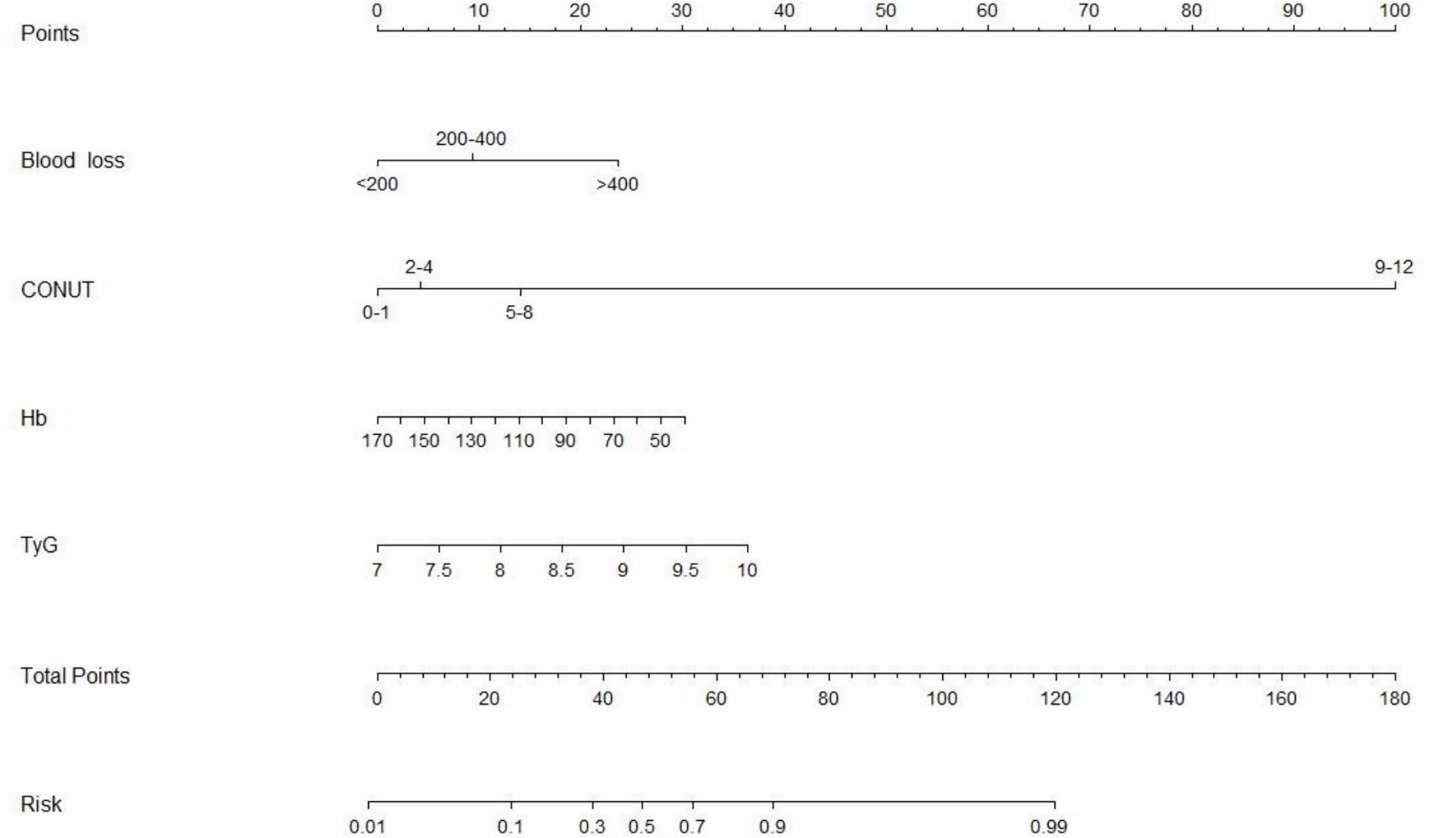
Nomogram predicting the probability of blood transfusion.

**Figure 8:**
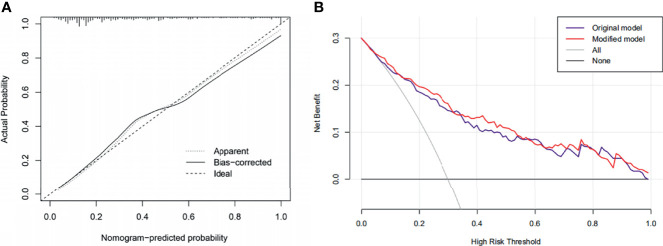
**(A)** Calibration curves of the nomogram. **(B)** Decision curve analysis (DCA) of the original and the modified model.

Based on the results mentioned above, we established a nomogram model using blood loss, CONUT, Hb, and TyG. By projecting the points corresponding to each variable to the “points” axis, the total scores were calculated to provide the corresponding prediction results (**Figure 7**). The discrimination power of the nomogram was appraised by the C-index. The C-index of the nomogram was 0.834. **Figure 8** shows the calibration curves of the cohort. The model demonstrated good consistency. The results of the decision curve analysis (DCA) for the blood transfusion nomogram before and after improvement were also presented, which suggested that the modified nomogram model had a considerable net clinical benefit that was not weaker than that of the original model.

The nomogram prediction model finally incorporated four predictors. The minimum calculated sample size was 365 cases, with 142 cases in the blood transfusion group. According to the sample size computation, the number of patients included in the present study was deemed sufficient. The incidence of blood transfusion in the present study was approximately 38.8%.

## Discussion

This study compared the short-term outcomes and economic costs of patients undergoing TG with or without blood transfusion. Simultaneously, potential variables for blood transfusion were analyzed, and the stapler used, surgical technique, preoperative Hb, blood loss, operation time, CONUT score, and the TyG index were further explored. A streamlined nomogram was created including blood loss, CONUT, Hb, and TyG by combining the previous results, the correlation analysis, and the importance analysis of the XGB algorithm. In addition, a comparison with the original model showed that the performance of the modified model was not significantly weaker. The nomogram showed good diagnostic performance (C-index = 0.834). An internal verification was conducted, and the calibration curves demonstrated good model consistency. The DCA also showed clinical benefits that were no worse than those of the original model. Moreover, all the indicators were easily obtained, which reduced the patients’ additional medical expenditure and medical behavior and made the application of the nomogram easier. Meanwhile, we found that potential preoperative nutritional indicators were incorporated, with an indispensable role in the nomogram. Thus, greater attention should be paid to the nutritional status of patients undergoing TG and nutritional intervention should be carried out, when necessary.

The effect of blood transfusion on the short- and long-term prognosis of patients undergoing gastric cancer surgery remains unclear. For some critically ill patients, especially those with unstable hemodynamics, blood transfusion is an important and a life-saving intervention (11, 12). On the contrary, research has shown a number of potential risks of blood transfusion, including allergic reactions, fever, hemolytic reactions, and volume overload. In addition, inflammatory reactions and blood transfusion usually worsen the prognosis of patients (13). Studies have also reported that patients are more likely to develop infections after blood transfusion, irrespective of clean or contaminated surgeries (14). There is also evidence that a patient’s immune function is affected as blood transfusion might affect the body’s immunosuppressive prostaglandins and the activity of heterogeneous T cells (15). In this study, the change in Hb was not pronounced in the transfusion group, but the elevated costs and the delay in discharge were significant. The seriousness of patients requiring blood transfusion and the complications after a blood transfusion may result in this outcome, indicating that blood transfusions should be carried out with caution as they are likely to lead to unpredictable and adverse prognoses.

The relationship between blood loss and blood transfusion was apparent. The greater extent of resection and the difficulty of anastomosis in TG make it more important to pay attention to refinements in order to reduce bleeding. Since the first report of laparoscopy-assisted distal gastrectomy (LADG) in 1994, the safety and the feasibility of laparoscopic surgery have been confirmed in continuous practice (16–18). Compared with open surgery, laparoscopic surgery can perform lymph node dissection in a clearer field of view, increasing the safety of the operation; although it extends the operation time, the reduction in bleeding is significant. At the same time, with advances in surgical technology, total laparoscopic TG has gradually become popular, and the indications have continuously expanded (19). Furthermore, intraoperative blood loss has also been found to be possibly related to long-term prognosis in previous studies (6, 20). The close relationship between blood loss and blood transfusion, as well as the risk of distant metastases that may arise from blood loss, is worth considering. Hematogenous metastasis of tumors and suppression of antitumor immunity have been reported to be possibly related to this (21–23). It is still recommended to perform quantitative tests for blood loss, which will help in reaching a more accurate conclusion.

It is quite common for patients with upper–middle tumors to be affected by diet, necessitating more attention to the nutritional status. The CONUT score is a method used to evaluate patients’ immune and nutritional status. It includes the serum albumin content, total cholesterol level, and the total number of peripheral blood lymphocytes, and the score is calculated based on the index content (24). Studies have shown that the CONUT score is closely related to the prognosis of various tumors (25). In gastric cancer, CONUT-related studies have focused on long-term prognosis (26–28). There are a few studies on the CONUT during hospitalization, but none of these studies found a relationship between CONUT and blood transfusion, so far. In terms of the indicators of composition, albumin is one of the most important references in the clinical assessment of the nutritional status of patients. Hypoproteinemia is often associated with anastomotic leaks, infections, and thoracoabdominal effusion (29–31). Total cholesterol level is often related to metabolism, antioxidant reserve, and inflammatory response (32–34). Lymphocytes are an important part of the human immune response system, helping to fight tumors by inhibiting the proliferation and migration of cancer cells (35). Taken together, the CONUT provides a comprehensive, easy-to-use scale enabling the assessment of the preoperative status.

Preoperative low Hb is the most important risk factor for perioperative blood transfusion (36). It was reported that severe anemia, Hb level of <9.0 g/dl, was associated with an increased odds of transfusion (37). Due to the characteristics of gastric cancer, many patients have anemia before surgery. In upper–middle and upper gastric cancers, in particular, obstruction is common, and the patient’s compromised diet combined with the depletion of the tumor further worsens the anemic symptoms. Surgeons are usually not aware of the severity of tumoral anemia due to insufficient attention, resulting in less effective intervention. There is also a lack of accurate assessment of the extent of intervention, resulting in wasted blood resources or inadequate intervention. It is common knowledge that Hb is usually the primary indicator for transfusion, but this study showed that the assessment of transfusion is still a multifactorial process that requires considering multiple preoperative and intraoperative factors in order to achieve maximum efficacy.

The TyG index is based on the study of triglyceride and insulin sensitivity in skeletal muscles and is calculated using fasting triglyceride and fasting blood glucose measurements. In addition, the latest research demonstrates that the TyG index is generally considered to be related to insulin resistance. Furthermore, several previous studies have explored the relationship between TyG and the occurrence and prognosis of various clinical diseases (38, 39). Our investigation suggests that an elevated TyG index increases the likelihood of blood transfusion preoperatively in patients undergoing TG. Obesity and inflammatory markers such as tumor necrosis factor-α and interleukin-6 were considered in relation to TyG; these factors can increase the difficulty of surgery and the possibility of blood transfusion (40, 41). There are also some reports on the relationship between TyG and diabetes and cardiovascular diseases, which may be related to vascular fragility, intraoperative hemostasis, and blood transfusion (42, 43). Furthermore, TyG has been reported to be a risk factor for non-alcoholic liver disease, influencing liver function and increasing the odds of blood transfusion. Recent research has shown that perioperative blood transfusion adversely affects the prognosis of patients; thus, more relevant studies are necessary to further clarify the comprehensive results of the correlation between the TyG index and blood transfusion and the long- and short-term prognosis. In brief, we attempted to demonstrate that an abnormally elevated TyG index has implications for blood transfusion, which provides new possible research directions to benefit patients.

The stapler used, the surgical technique, and the operation time had effects on perioperative blood transfusion. However, after further exploration, they were found to be less likely relevant. The choice of a circle stapler (CS) or a linear stapler (LS) is still worth investigating. CS is more commonly used in LADG and open surgery, while LS is more common in total laparoscopic surgery. LS is superior in terms of the size of anastomosis and the requirements for tunnels, while CS is more familiar to most clinicians. In addition, LS requires a higher esophageal separation in TG, which is very difficult in patients with huge tumors or obesity and may necessitate a blood transfusion. Furthermore, previous studies have reported a correlation between the increased duration of operation and blood transfusion (44). A longer surgery time usually means more difficulties encountered during surgery and a longer exposure time. A longer operative time has been demonstrated to be directly related to complications and reoperation (45).

Currently, there are requirements for blood transfusion; however, considering the lag of laboratory tests and the particularity of clinical changes, transfusion is still determined by the surgeon based on the patient’s condition (46). With the effects of the COVID-19 pandemic, the number of blood donors dropped significantly. In the case of uncertain prognosis and a shortage of blood resources, blood transfusion should be carried out more cautiously for safety and the full use of blood resources. In this study, combined with surgery and basic conditions, it was found that some surgical options and the nutritional status are risk factors for patients with blood transfusion. When conflicts arise, the surgery can be delayed to actively prepare blood resources, formulate a more appropriate surgical plan, or provide nutritional support for patients to reduce the possibility of blood transfusion. In particular, adjusting the patient’s preoperative general status is noteworthy in order to reduce the likelihood of transfusions and avoid a lack of blood resources that could lead to poor outcomes.

The present study comprehensively analyzed the prognostic factors for blood transfusion. Machine learning was used to establish a modified, accurate, and convenient nomogram prognostic model. However, the study still has some limitations. Firstly, this study was retrospective, and some unknown factors will inevitably lead to bias. Secondly, this was a single-institution study, and some patients were excluded for various reasons, possibly affecting the generalizability of this model. Therefore, more prospective, multi-institutional studies should be considered. In addition, the exact rationale for these, such as total cholesterol, lymphocyte count, and TyG with blood transfusion, has not been elucidated, and more basic research is worthwhile.

In conclusion, a nomogram was established and modified to predict the need for blood transfusion in patients undergoing TG. The exploratory discovery of the relationship between CONUT and TyG and blood transfusion provides a basis for further research. The nomogram is useful in guiding the surgeon’s decision regarding blood transfusion, timely nutritional intervention, and making full use of clinical blood resources.

## Data Availability Statement

The original contributions presented in the study are included in the article/**Supplementary Material**. Further inquiries can be directed to the corresponding author.

## Ethics Statement

The studies involving human participants were reviewed and approved by the Ethics Committee of the First Affiliated Hospital of Soochow University. The patients/participants provided written informed consent to participate in this study.

## Author Contributions

All authors contributed to the study conception and design. Material preparation, data collection, and analysis were performed by JZ and LJ. The first draft of the manuscript was written by JZ and XZ. All authors commented on the manuscript. All authors contributed to the article and approved the submitted version.

## Conflict of Interest

The authors declare that the research was conducted in the absence of any commercial or financial relationships that could be construed as a potential conflict of interest.

## Publisher’s Note

All claims expressed in this article are solely those of the authors and do not necessarily represent those of their affiliated organizations, or those of the publisher, the editors and the reviewers. Any product that may be evaluated in this article, or claim that may be made by its manufacturer, is not guaranteed or endorsed by the publisher.

## Abbreviations

TG, total gastrectomy; PSM, propensity score matching; GC, gastric cancer; CONUT, control nutritional status; Hb, hemoglobin; TyG, triglyceride–glucose; C-index: concordance index; DCA, decision curve analysis; BMI, body mass index; PNI, prognostic nutritional index; ALB, albumin; LR, logistic regression; Tree, decision tree learning; XGB, extreme gradient boosting; RF, random forest; GBDT, gradient boosting decision tree; GBM, light gradient boosting machine; AUC, area under the curve; ROC, receiver operating characteristic; LADG, laparoscopy-assisted distal gastrectomy; CS, circle stapler; LS, linear stapler.
