# Supplementary material for: A Machine Learning-Modified Novel Nomogram to Predict Perioperative Blood Transfusion of Total Gastrectomy for Gastric Cancer
Source: Front Oncol. 2022 Apr 11;12:826760. doi: 10.3389/fonc.2022.826760 (PMC9035891; doi:10.3389/fonc.2022.826760)
Supplement: Supplementary file 1 [file DataSheet_1.pdf]

Supplementary Material

Supplementary Figure 1

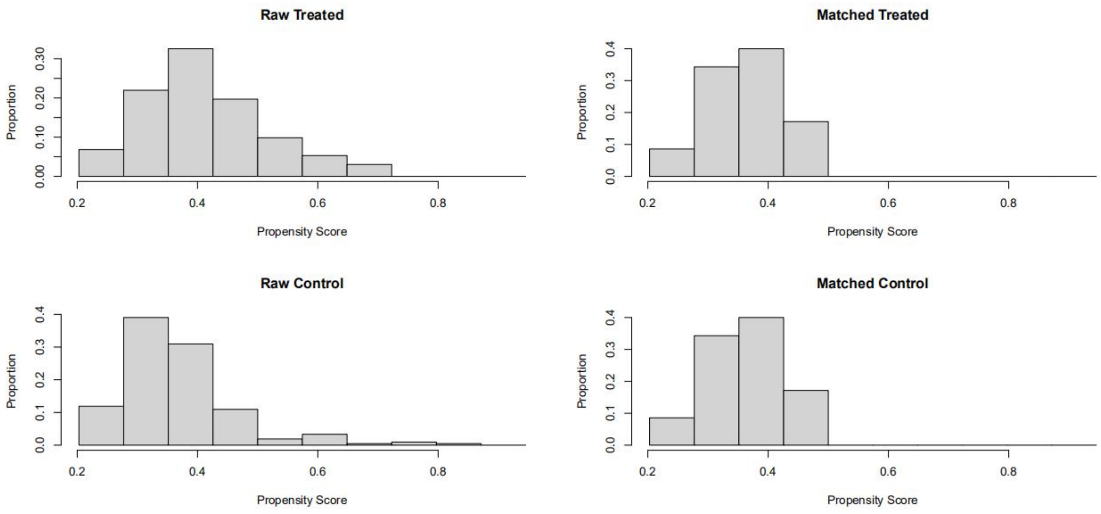

Supplementary Figure 2

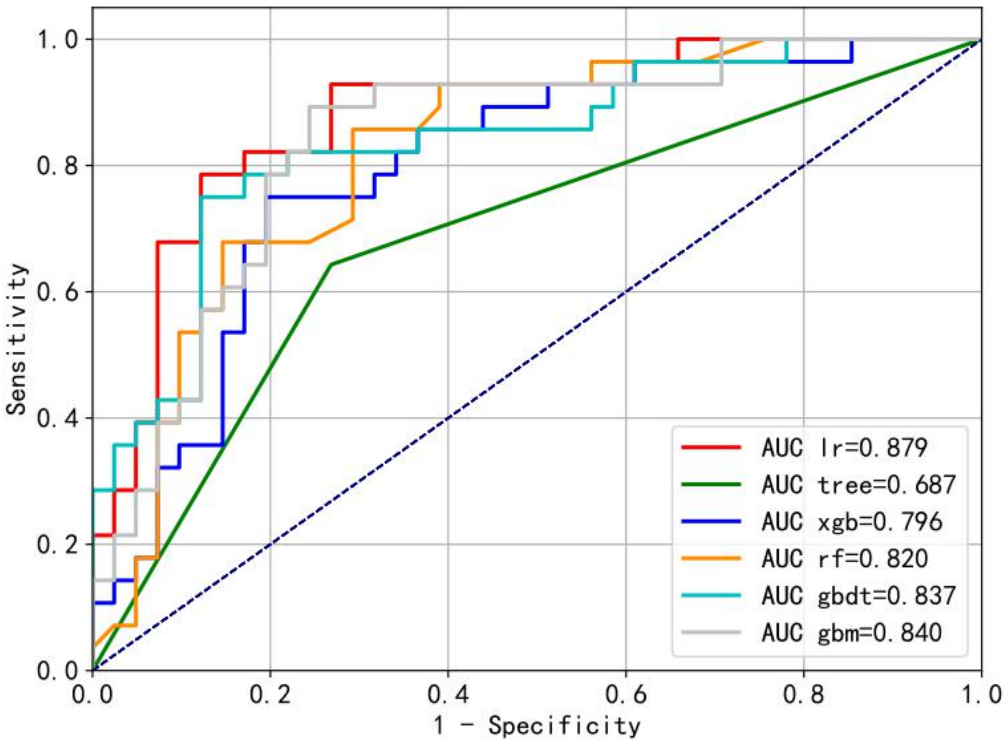

Supplementary Figure 3

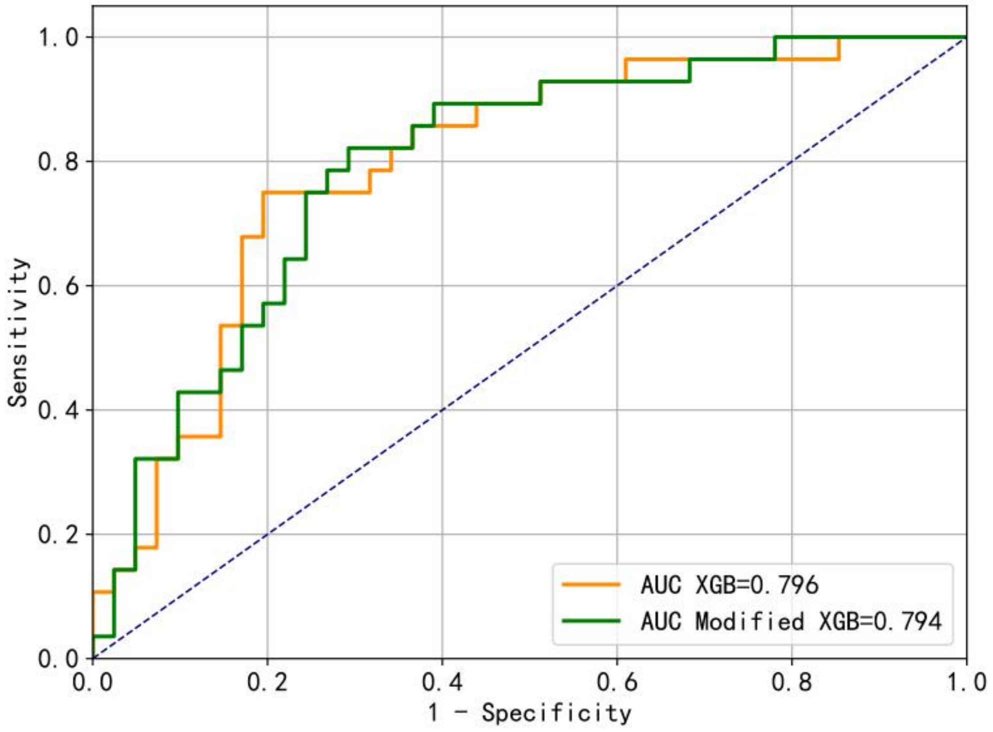

Figure legends

Supplementary Figure 1. After PSM, the score distribution is consistent and demonstrates a good match.

Supplementary Figure 2. ROC of different machine learning algorithms predict the blood transfusion.

Supplementary Figure 3. ROC of XGB and modified XGB.
